# ATM Release at Resected Double-Strand Breaks Provides Heterochromatin Reconstitution to Facilitate Homologous Recombination

**DOI:** 10.1371/journal.pgen.1003667

**Published:** 2013-08-01

**Authors:** Verena Geuting, Christian Reul, Markus Löbrich

**Affiliations:** Darmstadt University of Technology, Radiation Biology and DNA Repair, Darmstadt, Germany; Duke University, United States of America

## Abstract

Non-homologous end-joining (NHEJ) and homologous recombination (HR) represent the two main pathways for repairing DNA double-strand breaks (DSBs). During the G2 phase of the mammalian cell cycle, both processes can operate and chromatin structure is one important factor which determines DSB repair pathway choice. ATM facilitates the repair of heterochromatic DSBs by phosphorylating and inactivating the heterochromatin building factor KAP-1, leading to local chromatin relaxation. Here, we show that ATM accumulation and activity is strongly diminished at DSBs undergoing end-resection during HR. Such DSBs remain unrepaired in cells devoid of the HR factors BRCA2, XRCC3 or RAD51. Strikingly, depletion of KAP-1 or expression of phospho-mimic KAP-1 allows repair of resected DSBs in the absence of BRCA2, XRCC3 or RAD51 by an erroneous PARP-dependent alt-NHEJ process. We suggest that DSBs in heterochromatin elicit initial local heterochromatin relaxation which is reversed during HR due to the release of ATM from resection break ends. The restored heterochromatic structure facilitates HR and prevents usage of error-prone alternative processes.

## Author Summary

Double-strand breaks (DSBs) are critical DNA lesions because they can lead to cell death or, which is even more devastating, the formation of genomic rearrangements. Cells are equipped with two main pathways to repair such lesions, homologous recombination (HR) and non-homologous end-joining (NHEJ). HR is an error-free process and completely restores the genetic information, whereas NHEJ has the potential to form genomic rearrangements. We have previously shown that the structure of the chromatin is one important factor which determines the choice between these two pathways, such that DSBs localizing to highly condensed heterochromatic regions are mainly repaired by HR and breaks in more open euchromatic DNA undergo repair by NHEJ. Here, we investigate this aspect of DSB repair pathway choice. We show that DSB end-resection, which channels DSB repair into the process of HR, counteracts the profound local relaxation which initially takes place at the break site and reconstitutes the heterochromatic structure. Cells which are genetically modified, such that they cannot reconstitute the heterochromatic structure at resected DSBs, fail to employ HR and instead repair heterochromatic DSBs by alternative NHEJ mechanisms. Thus, chromatin modifications which occur during the process of end-resection prevent error-prone repair pathways from generating genomic rearrangements.

## Introduction

DNA double-strand breaks (DSBs) are among the most deleterious cellular lesions since they threaten genomic integrity and cell viability. To counteract cell degeneration and to preserve genomic integrity, a complex network of DSB repair and signaling processes has evolved [1]–[4].

Two main DSB repair pathways exist, canonical non-homologous end-joining (c-NHEJ) and homologous recombination (HR) [5], [6]. In mammalian cells, c-NHEJ represents the major repair pathway for ionizing radiation (IR)-induced DSBs [7]. C-NHEJ repairs unresected break ends without the need for sequence homologies and can function throughout the cell cycle [8]. The key factors in c-NHEJ involve the KU70/80 heterodimer, which binds to the DSB end, and the DNA-dependent protein kinase catalytic subunit (DNA-PKcs), which, together with KU70/80, constitutes the DNA-PK holoenzyme. The repair process is completed by a complex of DNA ligase IV, XRCC4, and XLF/Cernunnos [5]. In contrast to c-NHEJ, HR is restricted to the S and G2 phases of the cell cycle where break ends undergo extensive resection and homologous DNA sequences on the sister chromatid serve as a template for repair. In addition to the repair of DSBs, HR functions during the S phase to restart stalled or collapsed replication forks [9]. HR is initiated by CtIP-dependent resection to create 3′-overhangs at the DSB ends [10], [11]. Following extended resection by EXO1 or BLM/DNA2, loading of RAD51 onto single-stranded DNA (ssDNA) is facilitated by BRCA2, XRCC2, and XRCC3. RAD54-mediated homology search then promotes strand exchange and Holliday junction formation [6]. HR is completed after repair synthesis by Holliday junction resolution and DNA end ligation. In the absence of c-NHEJ factors, DSB repair can also occur by an alternative end-joining mechanism, termed alt-NHEJ [12], [13]. In contrast to c-NHEJ but similar to HR, alt-NHEJ involves CtIP-dependent resection. The resected break ends are subsequently rejoined by a process involving micro-homologies and various repair factors such as poly (ADP-ribose) polymerase (PARP), DNA ligase I or III, and XRCC1 [14]–[17]. Although alt-NHEJ can efficiently operate in cells devoid of c-NHEJ factors, little is known about its ability to compensate for HR defects.

It has become clear over the last years that higher order chromatin structure impacts on the response to DSBs [18]. Thus, IR-induced DSBs in densely compacted heterochromatin (HC) are more difficult to repair than euchromatic (EC) DSBs and they require additional structural changes in the surrounding chromatin [19], [20]. One example are ATM-mediated chromatin changes due to KAP-1 phosphorylation [21]. In undamaged cells, KAP-1 forms HC by recruiting HP1, CHD3 and other remodeling factors [22], [23]. DSB-induced KAP-1 phosphorylation leads to release of CHD3 which locally relaxes HC and facilitates repair [23]. Other studies involving HP-1 mobilization have observed either a release from [24] or a recruitment to damaged chromatin [25]–[27]. These apparently conflicting findings have led to the suggestion that a transient release might be followed by an accumulation of HP1 at sites of DNA damage [19], [28]. However, it is often unclear how the various processes of chromatin modification impact on DSB repair and if different repair pathways are differentially affected.

Repair kinetics for IR-induced DSBs are biphasic, exhibiting a fast and a slow component [29]. The slow component accounts for the repair of a subset (15–20%) of IR-induced DSBs that are localized to HC DNA regions, whereas DSBs induced in EC regions are typically repaired with fast kinetics. In G1 phase, the fast and the slow component of DSB repair comprise a c-NHEJ mechanism [29]. ATM-dependent phosphorylation of KAP-1 on serine 824 (S824) is specifically required for the slow component [30], [31]. In G2 phase, in contrast, c-NHEJ accounts only for the fast DSB repair process, while the slow ATM-dependent HC component represents HR [32]. Thus, in G2, defined DSB populations, EC vs. HC breaks, are repaired by either c-NHEJ or HR, respectively. Despite the existence of two repair pathways in G2, a mutation in one of them leads to elevated unrepaired DSBs. Thus, c-NHEJ and HR cannot compensate for each other which might be attributed to the fact that c-NHEJ is unable to repair DSBs which have undergone extensive resection. Consistent with this notion, c-NHEJ can compensate for HR if resection is prevented by CtIP depletion [33]. What remains unclear is why alt-NHEJ, which in principal is able to rejoin resected break ends, cannot compensate for a loss of down-stream HR factors such as BRCA2 or RAD51.

In the present study, we analyzed the process of HR at HC DSBs in G2 phase. We show that the intensity of phosphorylated ATM at DSBs decreases during the process of resection, suggesting that ATM initially binds to but is then released from DSBs which undergo repair by HR. Consistent with this notion, chemical inhibition of ATM prior to but not after resection causes a repair defect. Thus, ATM has an early role during HR but is dispensable for later stages. This contradicts the situation in G1 where continuous ATM activity is required for HC DSB repair by c-NHEJ [34]. In G1, ATM functions to phosphorylate KAP-1, leading to its inactivation and local relaxation of the HC structure [30]. Moreover, depletion of KAP-1 by siRNA overcomes the requirement for ATM in G1 but leads to reduced HR usage in G2. Finally, following KAP-1 siRNA or expression of a phospho-mimic form of KAP-1, both of which cause HC relaxation, resected DSBs can be repaired by a PARP-dependent alt-NHEJ process. Together, these data show that the HC structure represents a barrier for repair by c-NHEJ and alt-NHEJ but facilitates usage of HR. ATM, which initially binds to DSBs, is released from break ends during the process of resection. This prevents usage of c-NHEJ and alt-NHEJ and commits resected DSBs to repair by HR.

## Results

### PhosphoATM (pATM) accumulation and activity is diminished at resected DSBs

**Figure 1:**
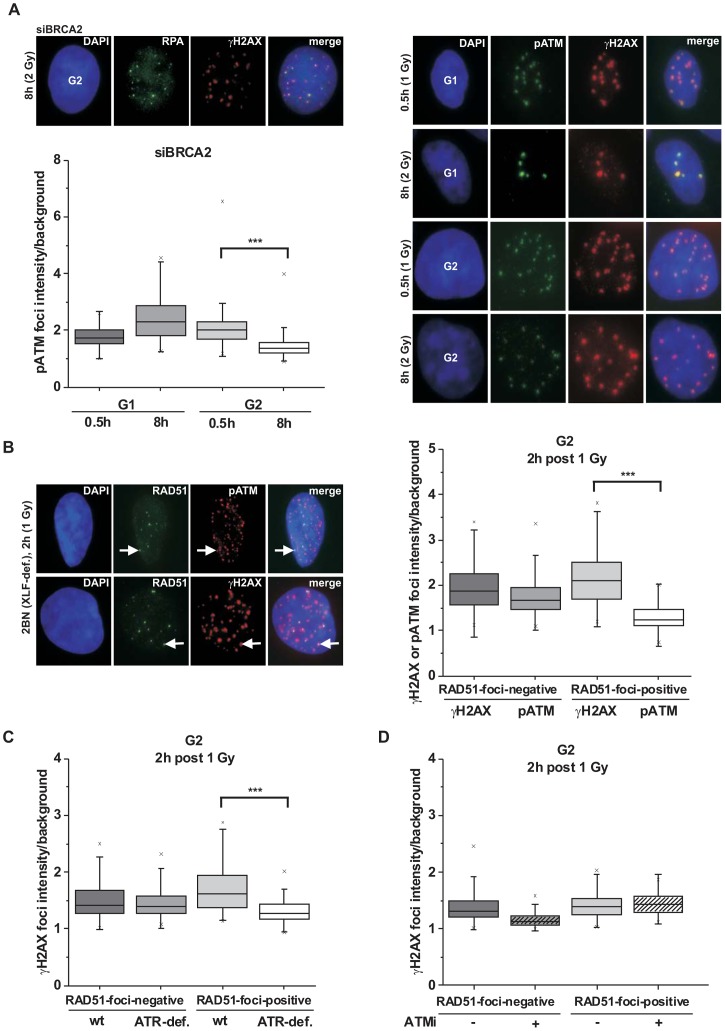
phosphoATM focal intensity decreases at DSBs undergoing resection. (**A**) A549 tumor cells treated with BRCA2 siRNA were irradiated with 1 Gy (0.5 h) or 2 Gy (8 h) and immunostained with the indicated antibodies. Using EdU and cell cycle markers to distinguish G1- from G2-phase cells [32], focal intensities of pATM were measured using ImageJ software (see Figure S1A). BRCA2 siRNA was used in this analysis to accumulate resected DSBs. (**B**) 2BN hTert (XLF-deficient) human fibroblasts were analyzed 2 h post IR with 1 Gy. Cells were stained against γH2AX and RAD51 or pATM and RAD51, and γH2AX or pATM focal intensities were measured at RAD51-foci-positive or RAD51-foci-negative foci. XLF-deficient cells were used in this analysis to prevent repair of EC DSBs during the time needed for resection of HC DSBs. (**C**) 82-6 hTert (wt) and F02-98 hTert (ATR-deficient) human fibroblasts were stained against γH2AX and RAD51 at 2 h post 1 Gy, and γH2AX focal intensities were measured as in (B). (**D**) 2BN hTert (XLF-deficient) human fibroblasts were stained against γH2AX and RAD51 at 2 h post 1 Gy, and γH2AX focal intensities were measured as in (B). Since both DNA-PK and ATM can phosphorylate γH2AX, cells were treated with DNA-PK inhibitor under all conditions. Inhibitors were added 1 h post IR, a time sufficient to allow for ATM-dependent resection and RAD51 loading (see Figure S1C). In (A–D), at least 300 foci from 3 independent experiments were analyzed for each point. Box plots were used with a maximum whisker-length of 1.5-fold the inter-quartile range; the lower and upper “x” indicates the 1% or 99% margin of the data range, respectively.

We have previously demonstrated that BRCA2-deficient cells exhibit elevated γH2AX foci levels at 8 h post irradiation in G2 [1], [32]. These unrepaired DSBs have undergone efficient end-resection as evidenced by RPA loading (Figure 1A) which might explain why they cannot be repaired by NHEJ. We sought to further characterize these breaks and observed that the pATM focal intensity in G2- but not in G1-phase cells is greatly diminished at 8 h compared with 30 min time points (Figure 1A and Figure S1A). In contrast, the γH2AX focal signal is equally intensive at 30 min and 8 h in G1 and G2 (Figure S1B). We also measured the pATM focal intensity at 2 h post IR, a time point when resected and unresected DSBs are present in G2-phase cells. Of note, the pATM focal intensity of RAD51-foci-positive resected breaks is reduced compared with RAD51-foci-negative unresected breaks. In contrast, the γH2AX focal intensity is similar or even slightly increased at resected versus unresected DSBs (Figure 1B). These findings suggest that the pATM focal intensity decreases during resection in G2. pATM contributes, together with DNA-PKcs and ATR, to the phosphorylation of H2AX [35], [36]. To test if the loss of pATM intensity at the break site leads to reduced ATM activity, we measured the γH2AX focal intensity in cells with strongly diminished levels of ATR, a kinase which is activated by ssDNA regions [37]. Significantly, although ATR-deficient cells show γH2AX focal intensities at unresected DSBs similar to wildtype (wt) cells, they exhibit greatly diminished intensities at resected breaks (Figure 1C). Consistent with the notion that ATM is active at unresected but not at resected DSBs, chemical inhibition of ATM only affects γH2AX foci intensities at unresected but not at resected DSBs (Figure 1D and Figure S1C).

**Figure 2:**
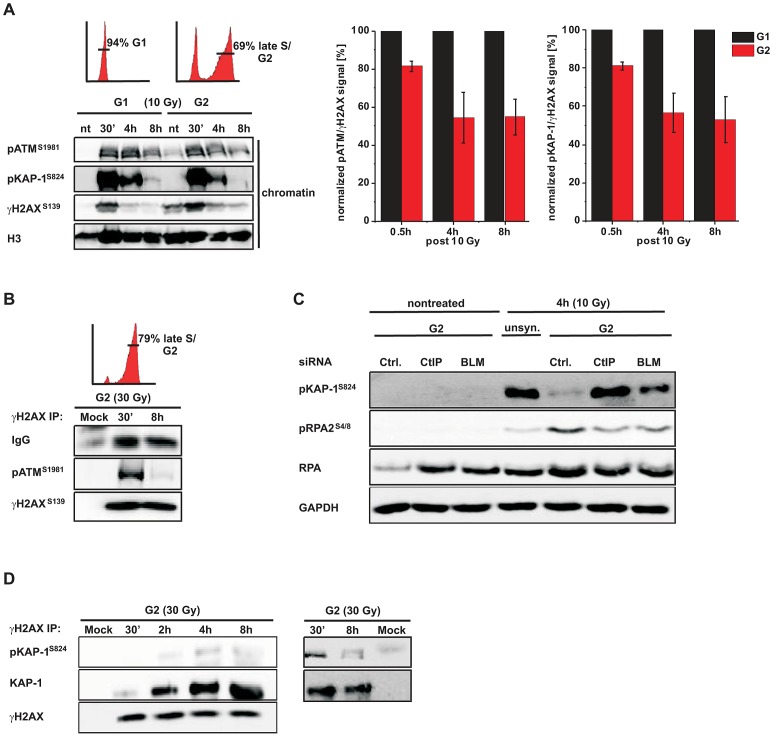
phosphoATM activity is diminished at resected DSBs. (**A**) G1-synchronized and G2-enriched A549 tumor cells were irradiated with 10 Gy and harvested at indicated time points. Fractionated chromatin (chromatin) was immunoblotted (left panel), and pATM, pKAP-1 and γH2AX levels of the chromatin fraction from the same blot were quantified using ImageJ. The right panels show the ratio of pATM or pKAP-1 relative to γH2AX for G1 and G2 cells at various time points. The data for G2 was normalized to G1 which was set to 100% (mean ± SEM from ≥2 experiments). (**B**) G2-synchronized HeLa tumor cells were irradiated with 30 Gy, harvested at the indicated time points, immunoprecipitated (IP) with γH2AX antibody and analyzed by immunoblotting. In G2 cells, pATM is co-immuno-precipitated with γH2AX at 30 min but not at 8 h post IR. The depicted FACs distributions in panels (A) and (B) represent the cell populations at the time of irradiation. How these populations change during repair incubation is shown in Figure S2. (**C**) HeLa tumor cells were treated with siRNA, synchronized in G2, and whole cell extracts were analyzed by immunoblotting 4 h post 10 Gy. pKAP-1 is detected in unsynchronized but not in G2-synchronized cells unless either CtIP or BLM is depleted. Depletion of CtIP or BLM did not affect the cell cycle distribution (see Figure S2C). (**D**) G2-synchronized HeLa tumor cells were irradiated with 30 Gy, harvested at the indicated time points, immunoprecipitated (IP) with γH2AX antibody and analyzed by immunoblotting. The level of KAP-1 co-immuno-precipitated with γH2AX increases with increasing repair time post IR. KAP-1 is substantially phosphorylated at early but not at later times.

We next sought to confirm the immunofluorescence (IF) measurements by Western blotting. We used A549 tumor cells which can be efficiently synchronized in G1 by serum starvation and moderately enriched in G2 by double thymidine blocking (Figure S2A). The level of chromatin-bound pATM decreases with time after IR due to ongoing repair in G1 and in G2 but, importantly, at later times the pATM level per γH2AX level is smaller in G2-enriched than in G1-synchronized cells (Figure 2A). We also measured pKAP-1 (S824) levels as a specific read-out for ATM activity [21] and obtained similar results (Figure 2A). We next wished to measure pATM bound to DSBs and employed immunoprecipitation (IP) experiments. For this, we used HeLa tumor cells which can be efficiently synchronized in G2 (Figure S2B). Strikingly, pATM bound to γH2AX is readily detected at 30 min but nearly absent at 8 h post IR in G2 (Figure 2B). To directly show that the diminished pATM activity in G2 is a result of resection, we inhibited resection by depleting CtIP or BLM [38] and measured pKAP-1 levels. G2-synchronized HeLa tumor cells show a strongly reduced pKAP-1 level at 4 h post IR compared with unsynchronized cells which is fully or partly restored after CtIP or BLM depletion (Figure 2C and Figure S2C). To provide evidence for the restoration of chromatin condensation at resected DSBs, we performed IP experiments as in Figure 2B. We observed that the level of KAP-1 bound to γH2AX continuously increases with repair time (Figure 2D), possibly due to an enrichment of HC DSBs at longer times and the recruitment of KAP-1 to damaged sites as previously reported [25]. Importantly, γH2AX-bound KAP-1 is substantially phosphorylated at early times post IR but largely unphosphorylated at later times (Figure 2D). Together, these biochemical approaches confirm the IF data above and provide strong evidence that ATM accumulation and activity is strongly reduced at DSBs which undergo resection. This leads to KAP-1 dephosphorylation and possibly the restoration of HC. The observed diminished ATM activity at resected DSBs is consistent with studies using a human cell extract-based assay in which ATM is activated by blunt DSB ends and ends with short ss overhangs but not by extended ssDNA regions which arise during the process of resection [39].

### ATM is dispensable for later stages of HR

**Figure 3:**
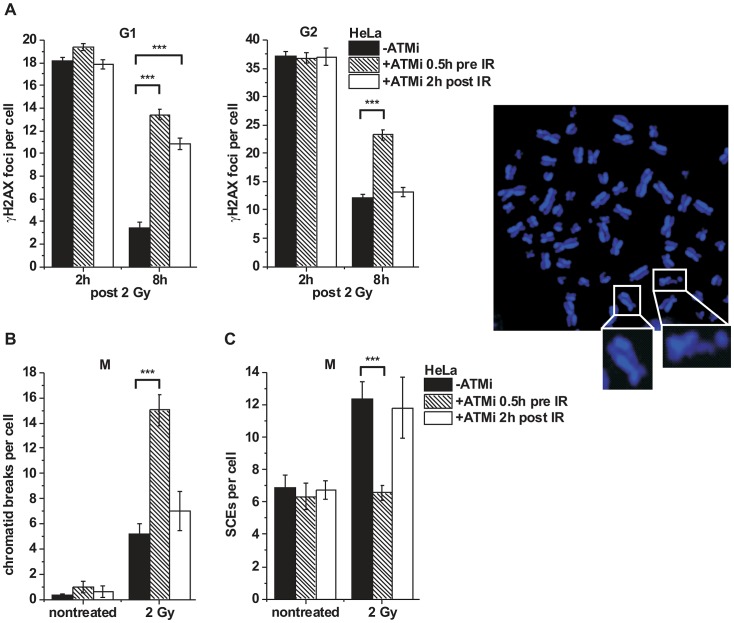
ATM is not required for later stages of HR. (**A**) γH2AX foci were analyzed in G1- and G2-irradiated A549 tumor cells as previously described [32]. Cells were treated with ATMi 0.5 h prior to or 2 h post IR to investigate the impact of ATM inactivation at various stages during repair. Background foci numbers were subtracted. At least 40 cells were analyzed per data point and experiment (mean ± SEM from ≥3 experiments). (**B–C**) Chromatid breaks (B) and SCEs (C) were analyzed in mitotic HeLa tumor cells at 8 h post 2 Gy. Cells were treated with caffeine and colcemid at 5 h post IR to abolish the G2 checkpoint and collect cells in mitosis. The addition of caffeine does not affect homologous recombination levels as assessed by SCE formation [42]. Cells were labeled with EdU, and only EdU-negative cells (i.e. cells in G2 at the time of irradiation) were included in the analysis. Cells were treated with ATMi as in (A). At least 40 metaphases were analyzed per data point and experiment (mean ± SEM from ≥3 experiments). Example of a DAPI-stained metaphase spread with an enlarged SCE (left panel) and chromatid break (right panel). *P* values were obtained by *t*-test and represent a comparison of all cells analyzed in the indicated cell populations (***: p<0.001).

ATM has been implicated in early steps of HR [33], [40], [41]. A prediction of our findings above is that ATM is no longer required for HR after resection has occurred. To test this, we inactivated ATM either before or at 2 h post IR, a time point when resection has occurred (Figure S1C), and investigated the efficiency of DSB repair. γH2AX foci numbers at 8 h post IR were substantially elevated both in G1- and G2-phase cells treated with ATM inhibitor (ATMi) before IR but only in G1-phase and not in G2-phase cells if ATMi was added 2 h post IR (Figure 3A). We also analyzed mitotic chromatid breakage in G2-irradiated cells and observed substantially elevated break levels if ATMi is administered before irradiation but not if it is added 2 h post IR (Figure 3B). HR in G2 leads to sister chromatid exchanges (SCEs) [42] which are diminished if ATM is inhibited before but not at 2 h after IR (Figure 3C). Together, these data show that ATM is dispensable for HR stages that occur after resection has taken place.

### KAP-1 depletion overcomes the BRCA2 repair defect

**Figure 4:**
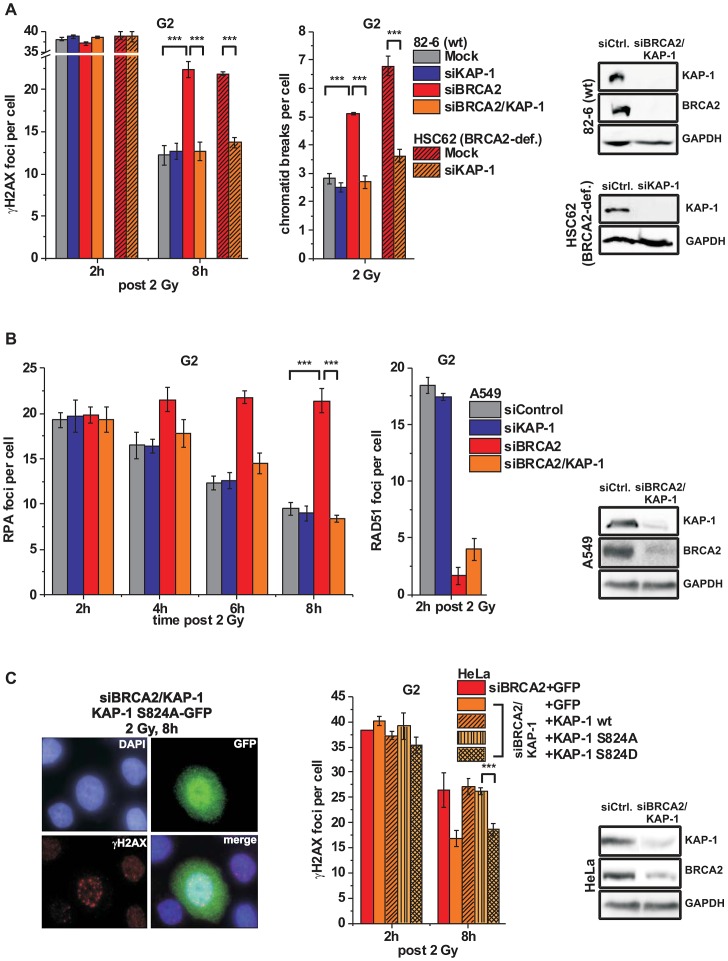
KAP-1 depletion allows HC DSB repair in the absence of BRCA2. (**A**) γH2AX foci and PCC analysis in G2-irradiated 82-6 hTert (wt) and HSC62 hTert (BRCA2-deficient) human fibroblasts. (**B**) RPA and RAD51 foci analysis in G2-irradiated A549 tumor cells. (**C**) Endogenous KAP-1 and BRCA2 was depleted in HeLa tumor cells by siRNA, and cells were transfected with GFP-tagged and siRNA-resistant empty (GFP), wt or mutated (phospho-mutant S824A or phospho-mimic S824D) KAP-1 plasmids. γH2AX foci were analyzed in GFP-positive G2-irradiated cells. EdU and cell cycle markers were used to distinguish G2- from S- and G1-phase cells [32]. In (A), (B) and (C), foci numbers or PCC breaks from unirradiated cells were subtracted. At least 40 cells or PCC spreads were analyzed per data point and experiment (mean ± SEM from ≥3 experiments). KAP-1 and BRCA2 depletion in this and subsequent experiments was highly efficient (>90% as assessed by Western blotting). *P* values were obtained by *t*-test and represent a comparison of all cells analyzed in the indicated cell populations (***: p<0.001).

It was previously shown that ATM operates in G1 by continuously phosphorylating KAP-1 at heterochromatic DSBs and that KAP-1 depletion overcomes the requirement for this ATM function [34]. Since ATM accumulation and activity is reduced at resected DSBs, we next asked if KAP-1 depletion might affect DSB repair in G2. KAP-1 siRNA did not alter γH2AX foci numbers in wt cells but strikingly rescued the repair defect in BRCA2 mutants and cells treated with BRCA2 siRNA (Figure 4A and Figure S3A). The same effect was observed in CHO cells deficient for the HR factor XRCC3 as well as in RAD51-depleted CHO cells (Figures S3B and S3C). Moreover, KAP-1 siRNA reduced the elevated level of chromatid breaks in BRCA2-deficient cells to that of wt cells (Figure 4A). We also measured the formation of SCEs and did not observe any IR-induced SCE formation in BRCA2/KAP-1-depleted cells (Figure S3D). Finally, we investigated cells containing an integrated HR reporter with two differentially mutated GFP genes [43]. Expression of the endonuclease I-SceI generates a DSB in one of the two genes which can be repaired by HR (gene conversion) with the second gene copy as a template, resulting in a cell with functional GFP. HR frequencies assessed by the fraction of GFP-positive cells are significantly decreased after BRCA2 depletion and dual depletion of BRCA2 and KAP-1, confirming that the repair events occurring in BRCA2/KAP-1-depleted cells do not represent HR (Figure S3E). A pathway switch from HR to c-NHEJ has recently been demonstrated for heterochromatic DSBs after the inhibition of resection by CtIP siRNA, consistent with the idea that resection determines DSB repair pathway choice [33]. Therefore, we asked if RPA foci formation, as a read-out for resection, is affected by KAP-1 depletion. Significantly, wt and BRCA2-depleted cells show the same initial level of RPA foci at 2 h post IR which is unaffected by KAP-1 siRNA. These RPA foci persist in BRCA2-depleted cells up to 8 h post IR consistent with their elevated γH2AX foci level. In contrast, RPA foci numbers decrease with time due to ongoing repair in wt and BRCA2-depleted cells treated with KAP-1 siRNA (Figure 4B and Figure S3F). We also investigated RAD51 loading at resected DSBs and observed normal RAD51 foci numbers after KAP-1 siRNA in wt but not in BRCA2-depleted cells (Figure 4B).

The finding that a BRCA2-independent process repairs resected DSBs after combined BRCA2 and KAP-1 siRNA suggests that the commitment for HR results from the loss of pATM at resected DSBs which is overcome by KAP-1 depletion. To consolidate this finding, we investigated DSB repair in cells treated with KAP-1 siRNA and complemented with siRNA-resistant KAP-1 constructs which were mutated at the ATM-dependent phosphorylation site on S824 [30]. The BRCA2 repair defect, which is rescued after KAP-1 siRNA, is restored after complementation with wt KAP-1 or with KAP-1 rendered unphosphorylatable by mutating serine at position 824 to alanine (S824A). Significantly, however, KAP-1 mutated to a phospho-mimic aspartate at position 824 (S824D) fails to restore the BRCA2 repair defect (Figure 4C). Thus, KAP-1 phosphorylation at the established ATM site 824 overcomes the commitment for HR and DSB repair in the absence of BRCA2 can proceed by an HR-independent process.

### Alt-NHEJ can function as a back-up pathway for HR

**Figure 5:**
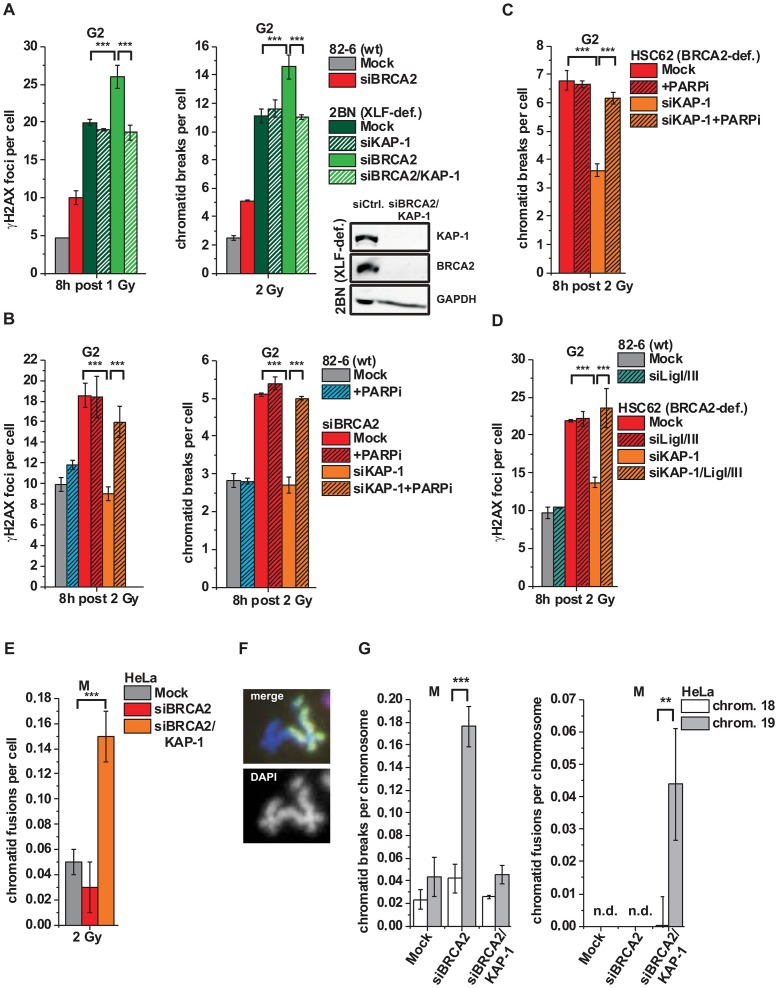
Alt-NHEJ can function as a back-up pathway for HR. (**A**) γH2AX foci and PCC analysis in G2-irradiated 82-6 hTert (wt) and 2BN hTert (XLF-deficient) human fibroblasts. (**B**) γH2AX foci and PCC analysis in G2-irradiated 82-6 hTert (wt) human fibroblasts treated with PARPi 0.5 h prior to IR. (**C**) PCC analysis from G2-irradiated HSC62 hTert (BRCA2-deficient) human fibroblasts treated with PARPi as in (B). (**D**) γH2AX foci analysis in G2-irradiated 82-6 hTert (wt) and HSC62 hTert (BRCA2-deficient) human fibroblasts. (**E–G**) Chromatid fusions and breaks in G2-irradiated mitotic HeLa tumor cells at 8 h post 2 Gy. Cells were treated with caffeine and colcemid at 5 h post IR to abolish the G2 checkpoint and collected in mitosis. Chromosomes were stained with Giemsa (panel E) or analyzed by FISH with probes specific for chromosomes 1 (red), 2 (green) and 4 (yellow) (panel F) or to chromosomes 18 and 19 (panel G). Foci numbers, chromatid breaks and fusions from unirradiated cells were subtracted. For panels A–E, at least 40 cells or 40 PCC/mitotic spreads were analyzed per data point and experiment (mean ± SEM from ≥3 experiments). For panel G, 50 mitotic spreads were analyzed per data point and experiment (mean ± SEM from ≥2 experiments). N.d. indicates that no chromatid fusions were observed under these conditions. *P* values were obtained by *t*-test and represent a comparison of all cells analyzed in the indicated cell populations (***: p<0.001).

Next, we wanted to investigate the process which is employed in BRCA2-deficient cells for the repair of resected DSBs. For this, we depleted BRCA2 and/or KAP-1 in cells deficient in the c-NHEJ factor XLF. XLF-defective cells show greatly elevated γH2AX foci and chromatid breaks consistent with the notion that c-NHEJ represents the predominant repair pathway in G2 [32]. Interestingly, depletion of BRCA2 leads to a similar increase in γH2AX foci/chromatid break numbers in wt cells and XLF mutants, demonstrating additivity of the two major repair pathways in G2, c-NHEJ and HR (Figure 5A). But most importantly in the present context, dual depletion of BRCA2 and KAP-1 did not affect γH2AX foci/chromatid break numbers in XLF mutants, demonstrating that the HR defect is rescued by KAP-1 depletion even in the absence of the c-NHEJ factor XLF (Figure 5A). The same effect was observed in CHO cells deficient in the c-NHEJ factor KU80 (Figure S4A).

We then tested if an alt-NHEJ pathway repairs DSBs in BRCA2/KAP-1-depleted cells and employed chemical inhibition of PARP (PARPi), a factor which has been implicated in alt-NHEJ [14], [17]. γH2AX foci and chromatid breaks were not significantly affected in wt cells treated with PARPi, demonstrating that alt-NHEJ processes do not contribute substantially to IR-induced DSB repair in normal cells. However, the elevated level of γH2AX foci/chromatid breaks in BRCA2-deficient cells, which is rescued after KAP-1 siRNA, is restored by PARPi (Figures 5B and 5C). Thus, PARPi precluded the repair events which arose in BRCA2-deficient cells after KAP-1 siRNA, demonstrating that a PARP-dependent process can function as a back-up pathway for HR. We also investigated other factors which have been described to function in alt-NHEJ. In CHO mutants deficient in XRCC1 as well as in cells deficient for DNA ligase I and III, KAP-1 failed to rescue the elevated γH2AX foci level which is conferred by a deficiency in BRCA2 or RAD51 (Figure 5D and Figure S4B). Consistent with the notion that alt-NHEJ can function as a back-up pathway for HR, we observed greatly increased levels of chromatid fusions in BRCA2/KAP-1-depleted cells (Figure 5E). To characterize the nature of these chromatid fusion events, we employed *fluorescence-in-situ-hybridization* (FISH) analysis with chromosome-specific probes. In one set of experiments, we used probes for chromosomes 1, 2 and 4 and observed that all fusion events (∼40 fusions from the analysis of ∼800 cells) occurred between heterologous chromosomes, that is, between a stained and an unstained chromosome or between two differently stained chromosomes (Figure 5F). Further, we employed probes for chromosome 19 which is exceptionally rich in KAP-1 binding sites and for the similar-sized chromosome 18 which is largely devoid of these sites [44]. Following BRCA2 depletion, we observed significantly higher breakage levels in chromosome 19 compared with chromosome 18, confirming that HR in G2 occurs mainly in KAP-1-dependent HC (Figure 5G). Importantly, following dual depletion of BRCA2 and KAP-1, chromosome fusions occur more often in chromosome 19 than in chromosome 18 confirming the notion that they arise from the misrejoining of chromatid breaks in KAP-1-dependent HC (Figure 5G).

### HR requires KAP-1-dependent heterochromatin

**Figure 6:**
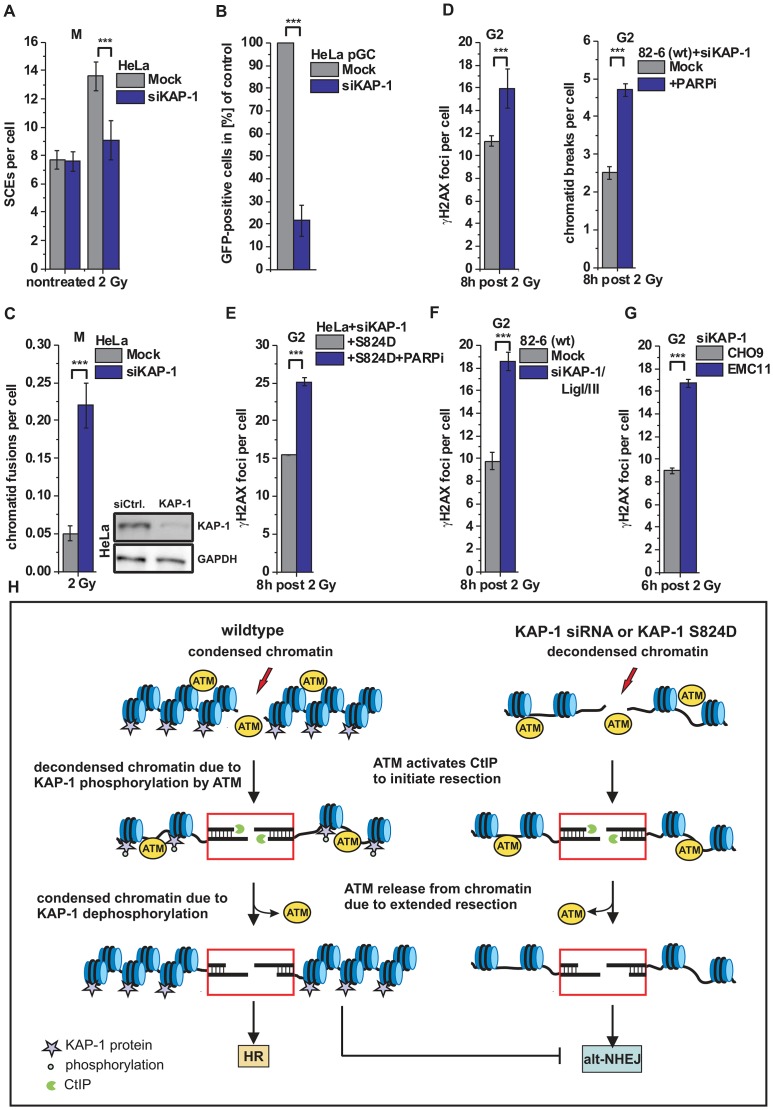
HR requires KAP-1-dependent heterochromatin. (**A**) SCEs in G2-irradiated mitotic HeLa tumor cells at 8 h post 2 Gy. Cells were treated with caffeine and colcemid at 5 h post IR to abolish the G2 checkpoint and collected in mitosis. (**B**) HR frequencies (gene conversion) after I-SceI expression in HeLa pGC cells carrying an integrated GFP reporter system. (**C**) Chromatid fusions analyzed from cells in panel A. (**D**) γH2AX foci and PCC analysis in G2-irradiated 82-6 hTert (wt) human fibroblasts. Cells were treated with PARPi 0.5 h prior to IR. (**E**) Endogenous KAP-1 was depleted in HeLa tumor cells by siRNA, and cells were transfected with GFP-tagged and siRNA-resistant phospho-mimic (S824D) KAP-1 plasmid. γH2AX foci were analyzed in GFP-positive G2-irradiated HeLa tumor cells treated with PARPi 0.5 h prior to IR. (**F**) γH2AX foci analysis in G2-irradiated 82-6 hTert (wt) human fibroblasts. (**G**) γH2AX foci analysis in G2-irradiated CHO9 (wt) and EMC11 (XRCC1-deficient) hamster cells. In (C–G), foci numbers, PCC breaks or chromatid fusions from unirradiated cells were subtracted. At least 40 cells or 40 PCC/mitotic spreads were analyzed per data point and experiment (mean ± SEM from ≥3 experiments). (**H**) Model of heterochromatic IR-induced DSB repair. In wt cells, ATM activates CtIP to initiate resection and phosphorylates KAP-1 to facilitate chromatin decondensation. Following extended resection, ATM is released from chromatin and KAP-1 is dephosphorylated, which likely results in restoration of condensed chromatin and a commitment to HR (left). In the case of decondensed chromatin due to KAP-1 depletion or expression of phospho-mimic KAP-1 (KAP-1 S824D), repair of resected DSBs occurs by alt-NHEJ, which is suppressed by chromatin condensation (right). *P* values were obtained by *t*-test and represent a comparison of all cells analyzed in the indicated cell populations (***: p<0.001).

The data above show that KAP-1 depletion allows heterochromatic DSBs to be repaired by an alt-NHEJ pathway in the absence of BRCA2, XRCC3 or RAD51. It is, however, unclear how the efficiency of HR in wt cells is affected by KAP-1-mediated chromatin changes. As shown above, γH2AX foci and chromatid breaks are repaired with similar kinetics with and without KAP-1 siRNA (see Figure 4A) but it is not known if repair after KAP-1 siRNA involves HR or, as in the case of HR mutants, an alt-NHEJ pathway. To address this question, we investigated the formation of SCEs in mitotic cells and observed greatly diminished SCE levels after KAP-1 siRNA in wt cells (Figure 6A). We also employed the HR reporter assay described above (Figure S3E) and observed strongly reduced HR levels following KAP-1 depletion (Figure 6B). Thus, KAP-1-depleted cells do not employ HR although repair occurs efficiently. We also analyzed chromatid fusion events as a read-out for incorrect end-joining. Strikingly, KAP-1-depleted cells show elevated chromosomal fusions, suggesting that the DSBs are repaired by an error-prone alt-NHEJ pathway (Figure 6C). This notion is consolidated by the observation that PARPi increases γH2AX foci and chromatid break numbers in cells depleted for KAP-1 or complemented with phospho-mimic KAP-1 (S824D) (Figures 6D and 6E). Further, cells deficient in DNA ligase I and III or in XRCC1 show elevated γH2AX foci levels following KAP-1 depletion (Figures 6F and 6G). Taken together, this data shows that HR is efficiently used in cells with unphosphorylatable KAP-1 and cannot occur if KAP-1 is depleted.

## Discussion

HR involves resection of DSB ends. Here, we investigated the process of HR at HC DSBs in G2 and showed that pATM, which initially binds to DSB ends, is released from the break sites during the process of resection. This leads to diminished KAP-1 phosphorylation at HC breaks and a commitment to repair such resected DSBs by HR. If the loss of KAP-1 phosphorylation is overcome by KAP-1 depletion or expression of phospho-mimic KAP-1, both of which are known to cause local HC relaxation, this commitment to HR is abolished and resected DSBs are repaired by an alt-NHEJ process. Thus, KAP-1-dependent HC facilitates later stages of HR whereas c-NHEJ and alt-NHEJ both require continuous HC relaxation due to ATM-dependent KAP-1 phosphorylation (see Figure 6H).

### ATM is released from resected DSBs

ATM binding and activation at DSB ends occurs within minutes after damage induction and is important for the initiation of various signaling processes [45]. Concomitant with the induction of signaling pathways, a variety of chromatin remodeling processes are initiated. This involves modifications which either relax or condense the chromatin structure in the surrounding of DSBs. However, it is currently unclear how these changes are chronologically orchestrated and how they differentially affect different DSB repair pathways in different chromatin compartments. Therefore, we focused our investigation on chromatin modifications which occur in HC regions due to the process of resection in order to specifically investigate how such chromatin changes impact on later stages of HR. We did not examine chromatin remodeling processes at early times which affect the decision to initiate resection.

We have previously shown that ATM is dispensable for the majority of DSB repair in G1 but that HC breaks strictly require ATM [30]. ATM's function during HC DSB repair in G1 involves continuous KAP-1 phosphorylation which leads to local HC relaxation [23]. Our finding that ATM is released from resected DSBs in G2 was therefore unexpected. However, there is precedence in the literature that ATM changes binding properties upon resection of DSBs. First, ATM's binding affinity to break ends has been reported to be attenuated with the progressive presence of ssDNA at resected DSBs [39]. This ATM attenuation is accompanied by increasing ATR activity [39], consistent with our result that H2AX phosphorylation at RAD51-foci-positive DSBs requires ATR. Second, 53BP1, a damage response factor which localizes to and facilitates pATM accumulation at DSB sites [34], has been reported to show reduced occupancy at resected DSBs in G2 [46]. Although the reported reduction of ATM accumulation and activity at resected breaks is consistent with published data, the functional consequence of this finding was hitherto unclear.

### ATM release at resected DSBs commits to HR

In G2 phase, DSB repair can be performed by NHEJ and HR. It is therefore remarkable that cells with mutations in BRCA2, XRCC3 or RAD51 exhibit unrejoined DSBs, which obviously are refractory to repair by NHEJ. Thus, it has been suggested that the process of resection commits DSB repair to HR and prevents usage of NHEJ [33]. Here, we provide mechanistic insight into the processes determining pathway usage upon resection. Since ATM is released from resected DSBs we reasoned that the concomitant reduction in KAP-1 phosphorylation prevents repair of resected breaks by NHEJ. Indeed, if loss of ATM-dependent KAP-1 phosphorylation is overcome by KAP-1 depletion or expression of phospho-mimic KAP-1, BRCA2-, XRCC3- or RAD51-deficient cells exhibit normal repair kinetics. Thus, it is not the resection *per se* but the loss of ATM activity at resected breaks which commits repair to HR.

### Alt-NHEJ can function as a back-up pathway for HR

HC DSBs which remain unrepaired in BRCA2-, XRCC3- or RAD51-deficient cells can be repaired if HC relaxation is provided by KAP-1 depletion or expression of phospho-mimic KAP-1. Interestingly, these DSBs undergo resection as evidenced by normal RPA foci formation. Thus, HC repair occurring in the absence of BRCA2, XRCC3 or RAD51 must involve a pathway which is capable of dealing with resected breaks. Consistent with the notion that alt-NHEJ can repair resected DSBs, we showed that the HC repair events occurring in the absence of BRCA2, XRCC3 or RAD51 require PARP, XRCC1 and DNA ligase I/III. We also observed that HC repair in the absence of BRCA2 has a significant propensity to lead to chromatid exchanges in G2-irradiated cells. Because alt-NHEJ has been implicated in the formation of genomic exchanges [47]–[50], this finding supports our contention that HC repair in the absence of BRCA2, XRCC3 or RAD51 involves alt-NHEJ.

### KAP-1-dependent heterochromatin facilitates HR

Perhaps surprisingly, we observed that the process of HR is nearly abolished in cells with depleted KAP-1, even in the presence of functional HR factors. This suggests that DSB repair pathway usage is significantly affected by chromatin modifications, favoring HR in condensed genomic regions. This notion is further supported by the observation that PARP inhibition or the loss of XRCC1 or DNA ligase I and III leads to elevated unrepaired breaks in KAP-1-depleted cells, which not only demonstrates that cells use alt-NHEJ but also, that they cannot employ HR in the absence of KAP-1-dependent HC. In summary, these findings establish that KAP-1-dependent HC is not only a barrier to repair by c-NHEJ or alt-NHEJ but, unexpectedly, also facilitates the process of HR.

Consistent with our results, depletion of HP1α or KAP-1 strongly reduces gene conversion frequencies in a I-SceI-based HR assay [25]. Furthermore, HP1α and KAP-1 is recruited to chromatin damaged by laser- or X-irradiation [26], [27] and depletion of HP1α diminishes SCE formation after treatment with camptothecin [51]. One explanation of how HC might promote HR is that a reduced spatial distance between sister chromatids in HC regions facilitates homology search [52]. In support of this idea, we have recently obtained preliminary evidence that the average distance between sister chromatids, measured by FISH analysis with locus-specific probes, is substantially larger in EC versus HC regions (Geuting et al., unpublished data). A similar mechanism has been suggested for cohesin proteins which might promote HR by providing the required proximity of sister chromatids in G2 phase [53]. Another explanation of how HC might facilitate HR is by suppressing alt-NHEJ processes. Although it is well established that the presence of KU70/80 at DSB ends prevents repair by alt-NHEJ, KU70/80 is likely released from resected DSB ends. Chromatin condensation occurring due to ATM release at resected DSBs might represent an alternative mechanism to keep error-prone alt-NHEJ processes in check.

### Conclusion

In conclusion, our study provides mechanistic insight into sequential events determining DSB repair pathway usage. First, we demonstrate that ATM activity is diminished at DSBs which undergo resection during the process of HR. Second, the concomitant loss of pKAP-1 at resected DSBs leads to local reconstitution of the HC superstructure and prevents repair of resected DSBs by alt-NHEJ. Thus, our study links two seemingly unrelated findings by showing how modifications at DSBs undergoing resection affect chromatin remodeling processes and DSB repair pathway usage.

## Material and Methods

### Cell lines and cell culture

Immortalized and transformed cell lines were 82-6 hTert (wt), HSC62 hTert (BRCA2-deficient, kindly provided by Dr. M. Digweed), 2BN hTert (XLF-deficient, kindly provided by Dr. P. Jeggo) and F02-98 hTert (ATR-deficient, kindly provided by Dr. P. Jeggo) human fibroblasts, HeLa-S3, HeLa pGC (kindly provided by Dr. J. Dahm-Daphi) and A549 human tumor cells, and CHO-AA8 (wt), IRS1SF (XRCC3-deficient; kindly provided by Dr. L. Thompson), CHO-K1 (wt), XRS6 (KU80-deficient, kindly provided by Dr. P. Jeggo), CHO-9 (wt) and EMC11 (XRCC1-deficient, kindly provided by Dr. B. Kaina) hamster cells. HeLa-S3 and A549 tumor cells were cultured in DMEM with 10% FCS and 1% NEAA; HeLa pGC cells additionally in 0.3 µg/ml puromycin. Human fibroblasts and CHO cells were cultured in MEM with 20% FCS, 1% NEAA. All cells were maintained at 37°C in a 5% CO_2_ incubator.

### RNA interference and plasmid transfection

SiRNA transfection was carried out with HiPerFect Transfection Reagent (Qiagen) following the manufacturer's instructions. siRNAs used in the experiments were: BLM (50 nM), Control (10 nM), CtIP (20 nM), KAP-1 (25 nM), RAD51 (20 nM), Lig I (20 nM), Lig III (20 nM) (Qiagen), and BRCA2 (25 nM) (SmartPool, Dharmacon). SiRNA sequences were: BLM (AAG CUA GGA GUC UGC GUG CGA), BRCA2 (GAA ACG GAC UUG CUA UUU A; GUA AAG AAA UGC AGA AUU C; GGU AUC AGA UGC UUC AUU A; GAA GAA UGC AGG UUU AAU A), Control (AAU UCU CCG AAC GUG UCA CGU), CtIP (UCC ACA ACA UAA UCC UAA UUU), KAP-1_A (CAG UGC UGC ACU AGC UGU GAG), KAP-1_B (CAU GAA CCC CUU GUG CUG UUU), RAD51 (AAG GGA AUU AGU GAA GCC AAA), Lig I (AAG GCA UGA UCC UGA AGC AGA), Lig III (AAC CAC AAA AAA AAU CGA GGA). Experiments were performed 48 h following siRNA transfection. For GFP-tagged siRNA-resistant KAP-1 plasmid transfection, HeLa tumor cells were incubated with KAP-1_B or KAP-1_B and BRCA2 siRNA and, 8 h later, transfected with 1 µg plasmid DNA using Lipofectamine LTX Transfection Reagent (Life Technologies). Cells were irradiated with 2 Gy, fixed and stained for γH2AX, EdU and GFP. Only GFP-positive cells were analyzed.

### Cell synchronization, X-irradiation and chemical treatment

A549 tumor cells were used for G1 synchronization and G2 enrichment. HeLa tumor cells were only used for G2 enrichment. G1 synchronization was carried out by 48 h serum starvation in DMEM without FCS and NEAA. 0.5 h before irradiation, medium was replaced by DMEM with FCS and NEAA. For G2 enrichment, a double thymidine blocking was used. Cells were blocked 16 h with 2 mM thymidine (Sigma), released in fresh medium for 9 h, blocked again with 2 mM thymidine for 16 h and released in fresh medium for 7–8 h. Synchronization was controlled by FACs analysis as described previously [54]. X-irradiation was performed at 90 kV and 19 mA with an aluminum filter (dose rate: 2 Gy/min). Chemical inhibitors were added 0.5 h prior to IR and maintained during repair incubation. The ATM inhibitor (Tocris KU 60019), the DNA-PK inhibitor Nu7441 (Tocris NU7026) and the PARP inhibitor PJ34 (Calbiochem PARP inhibitor VIII PJ34) were used at concentrations of 5 µM, 10 µM and 20 µM, respectively. Repair incubation was limited to time periods which provided that the majority of G2-irradiated cells remained in G2 (controlled by FACs analysis).

### Immunofluorescence

Cells were grown on glass coverslips. EdU (10 µM) was added 0.5 h prior to IR to discriminate between S- and G2-phase cells. In experiments analyzing G1-phase cells, nocodazol (100 ng/ml) was added 0.5 h prior to IR to prevent G2-phase cells progressing into G1 during repair incubation [55]. Cells were fixed and stained as described [56] and additionally stained with Click-it EdU (Life technologies). Antibodies used were: mouse-α-γH2AX at 1∶2000 (Millipore); rabbit-α-γH2AX at 1∶2000 (Abcam), mouse-α-pATM at 1∶1000 (Biomol), rabbit-α-RAD51 at 1∶15000 (Abcam), mouse-α-RPA at 1∶2000 (Neomarkers) and mouse-α-GFP at 1∶200 (Roche). Cells were analyzed with a Zeiss microscope and Metafer software (Metasystems). Samples were evaluated in a blinded manner. Foci intensities were analyzed using ImageJ software (see Figure S1A).

### HR reporter assay

HeLa pGC cells were incubated with siRNA and, 24 h later, transfected with 3 µg pBL464-pCBASce plasmid DNA using MaTra transfection (IBA). After 24 h, cells were again siRNA treated and, 48 h later, fixed and stained. 10000 cells per sample were analyzed with a Zeiss microscope and Metafer software (Metasystems).

### Protein extracts, chromatin fractionation and chromatin immunoprecipitation

Whole cell extracts were prepared as described [56]. For chromatin fractionation, cells were resuspended two times in NP-40 buffer (10 mM Tris/HCl pH 7.5, 10 mM NaCl, 3 mM MgCl_2_, 30 mM sucrose, 0.5% NP-40, 0.2 mM sodiumvanadate, 0.5 mM PMSF) and centrifuged for 10 min at 1500× g. Cell pellet was resuspended in Glycerol buffer (20 mM Tris/HCl pH 7.9, 100 mM KCl, 0.2 mM EDTA, 20% glycerol, 0.2 mM sodiumvanadate, 0.5 mM PMSF) and incubated 10 min on ice. After centrifugation (10 min, 1500× g) chromatin fraction was lysed and sonicated in RIPA buffer (50 mM Tris/HCl pH 8, 150 mM NaCl, 0.5 Na-deoxycholate, 1% Triton, 0.1% SDS). For immunoprecipitation, cells were fixed with 3% paraformaldehyd containing 2% sucrose for 5 min at 4°C, immediately washed with PBS, scraped in medium and centrifuged for 10 min at 400× g. Cells were resuspended two times in NP-40 buffer containing 15 mM caffeine and centrifuged for 10 min at 1500× g. Cell pellet was resuspended in equal volume Nuclease buffer (10 mM HEPES pH 7.5, 10 mM KCl, 1 mM CaCl_2_, 1.5 mM MgCl_2_, 0.34 M sucrose, 10% glycerol, 0.1% Triton-X-100, 0.2 mM sodiumvanadate, 0.5 mM PMSF, 15 mM caffeine), micrococcal nuclease (500 U/ml) was added and suspension was incubated for 45 min at 37°C. Equal volume of Solubilization buffer (2% NP-40, 2% Triton-X-100, 600 mM NaCl in Nuclease buffer) was added before mixing, brief sonication and clearifing for 10 min at 8000× g. Dynabead Protein G (Invitrogen) were blocked 1 h with 100 µg/ml salmon sperm DNA in 0.1% BSA/PBS and antibodies (4 µg) were linked to the beads, washed two times in 0.1% BSA/PBS and then incubated with the cell extract at 4°C over night. Beads were washed three times in Wash buffer (equal volume of Nuclease buffer and Solubilization buffer) and boiled in 2× Laemmli buffer for 5 min at 95°C.

### Immunoblotting

Western blotting was carried out at 300 mA for 1 h or at 80 mA over night. Nitrocellulose membrane (Roth) was blocked for 1 h in 5% low fat milk or 5% BSA in TBS/0.1% Tween20. Antibody incubation was carried out in TBS/0.1% Tween20/1% low fat milk or 5% BSA over night at 4°C, followed by HRP-conjugated secondary antibody incubation in PBS/0.1% Tween20/1% low fat milk or 5% BSA for 1 h. Immunoblots were developed using ECL (Roche). Signal detection was carried out with a chemi-smart-system (Vilber Lourmat). Primary antibodies used were: rabbit-α-pATM at 1∶1000 (Epitomics); rabbit-α-pKAP-1 (S824) at 1∶10000 (Epitomics); rabbit-α-KAP-1 at 1∶1000 (abcam); mouse-α-BRCA2 at 1∶1000 (Cell signaling); rabbit-α-GAPDH at 1∶1000 (Santa Cruz); mouse-α-γH2AX at 1∶1000 (Millipore); mouse-α-H3 at 1∶1000 (abcam); mouse-α-RPA2 at 1∶1000 (Calbiochem); rabbit-α-pRPA2 (S4/8) at 1∶10000 (Bethyl).

### Chromosomal analysis

EdU (10 µM) was added 0.5 h prior to IR and maintained to discriminate between S- and G2-phase cells. PCCs were harvested at 8 h, mitotic cells for SCE or FISH analysis between 5–8 h after IR as described [32]. Microscope slides were stained with DAPI (0.2 µg/ml) and Click-it EdU. For FISH analysis, whole chromosome probes for chromosomes 1, 2, and 4 or for chromosomes 18 and 19 were used (Metasystems). Chromosome spreads were recorded by Metafer software (Metasystems). Only EdU-negative chromosome spreads were analyzed.

## Supporting Information

Figure S1 (**A**) Measurement of foci and background intensities in a maximum intensity projection of a cell. Foci were identified by eye and foci shapes were defined by a region of interest (ROI) which was kept constant for all foci of the same experiment (upper panels on the left). The average pixel intensity (grey value) inside an ROI was taken to represent the focus intensity. The background was measured for each cell individually (cell shapes were determined by DAPI staining). For this, the most frequent (modal) grey value of the respective cell was determined which provided nearly identical results to the average grey value of the region without foci (see histogram on the right). The foci intensities were then normalized to the background intensity of the respective cell to account for variations in staining efficiency between different cells and samples. (**B**) A549 tumor cells treated with BRCA2 siRNA were irradiated with 1 Gy (0.5 h) or 2 Gy (8 h), immuno-stained as in Figure 1A, and focal intensities of γH2AX were measured using ImageJ software. (**C**) RAD51 foci were analyzed in G2-irradiated A549 tumor cells. Cells were treated with ATMi 0.5 h prior to or 1 h post IR. Foci numbers from unirradiated cells were subtracted. At least 40 cells were analyzed per data point and experiment (mean ± SEM from ≥3 experiments). *P* values were obtained by *t*-test and represent a comparison of all cells analyzed in the indicated cell populations (***: p<0.001).
(PDF)

Figure S2 (**A**) Cell cycle distributions of A549 tumor cells after synchronization in G1-phase by serum starvation (upper panels) or enrichment in G2 phase by double thymidine blocking (lower panels). (**B**) Cell cycle distributions of HeLa tumor cells after synchronization in G2 phase by double thymidine blocking (upper panels) or without synchronization (lower panels). (**C**) Cell cycle distributions of HeLa tumor cells after treatment with either CtIP or BLM siRNA and synchronization in G2 phase by double thymidine blocking.
(PDF)

Figure S3 (**A**) γH2AX foci were analyzed in G2-irradiated A549 tumor cells. (**B, C**) γH2AX foci were analyzed in G2-irradiated AA8 (wt) and IRS1SF (XRCC3-deficient) (panel B) or K1 (wt) (panel C) CHO cells. In samples treated with RAD51 siRNA, only RAD51-foci-negative cells were analyzed. (**D**) SCEs in G2-irradiated mitotic HeLa tumor cells at 8 h post 2 Gy. Cells were treated with caffeine and colcemid at 5 h post IR to abolish the G2 checkpoint and collected in mitosis. (**E**) HR frequencies (gene conversion) after I-SceI expression in HeLa pGC cells carrying an integrated GFP reporter system. (**F**) RPA foci were analyzed in G2-irradiated 82-6 hTert (wt) human fibroblasts. Foci numbers from unirradiated cells were subtracted. At least 40 cells were analyzed per data point and experiment (mean ± SEM from ≥3 experiments). *P* values were obtained by *t*-test and represent a comparison of all cells analyzed in the indicated cell populations (***: p<0.001).
(PDF)

Figure S4 (**A**) γH2AX foci were analyzed in G2-irradiated XRS6 (KU80-deficient) CHO cells. (**B**) γH2AX foci were analyzed in G2-irradiated CHO9 (wt) and EMC11 (XRCC1-deficient) CHO cells. In samples treated with RAD51 siRNA, only RAD51-foci-negative cells were analyzed. Foci numbers from unirradiated cells were subtracted. At least 40 cells were analyzed per data point and experiment (mean ± SEM from ≥3 experiments). *P* values were obtained by *t*-test and represent a comparison of all cells analyzed in the indicated cell populations (***: p<0.001).
(PDF)
