# Supplementary figures and images for: ATM Release at Resected Double-Strand Breaks Provides Heterochromatin Reconstitution to Facilitate Homologous Recombination
Source: PLoS Genet. 2013 Aug 1;9(8):e1003667. doi: 10.1371/journal.pgen.1003667 (PMC3731223; doi:10.1371/journal.pgen.1003667)

**A**

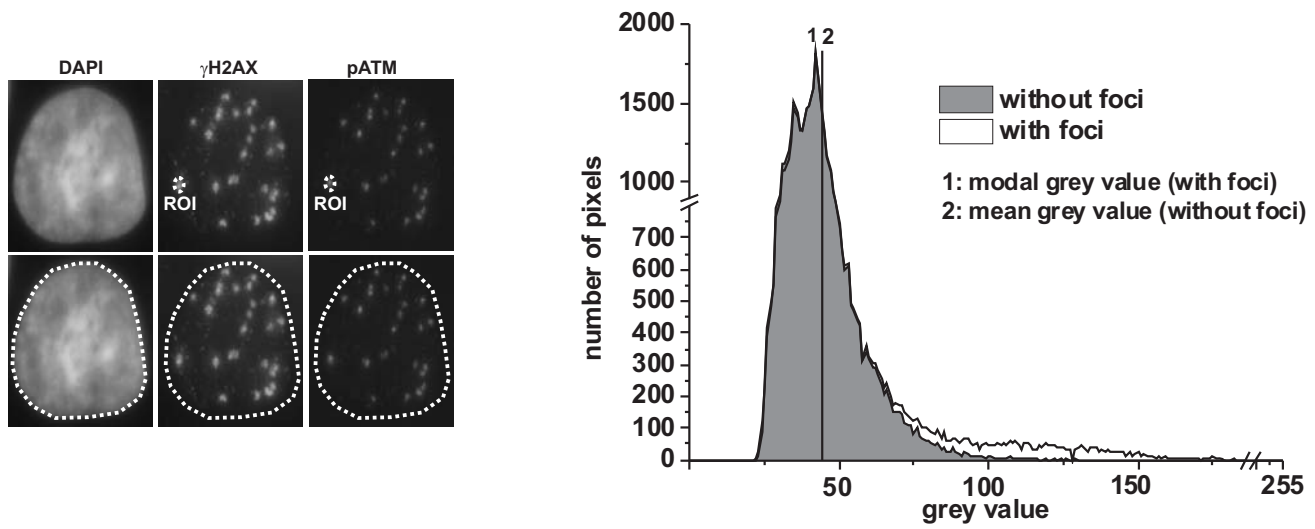

**B**

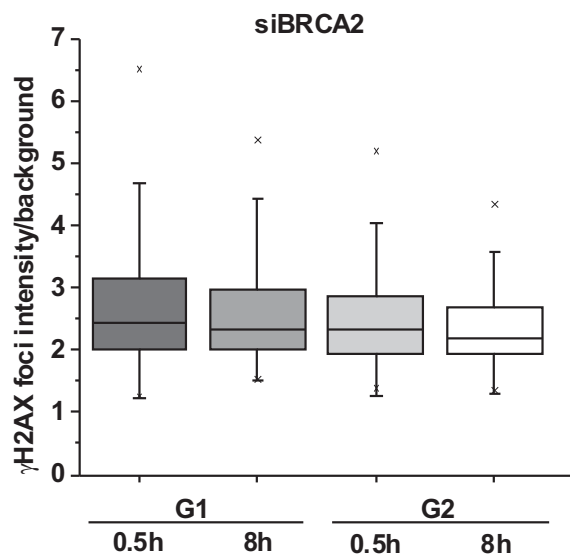

**C**

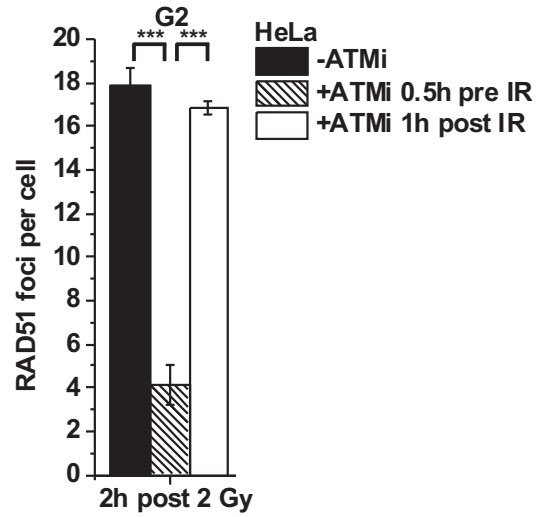

Supplement: Figure S1 — (A) Measurement of foci and background intensities in a maximum intensity projection of a cell. Foci were identified by eye and foci shapes were defined by a region of interest (ROI) which was kept constant for all foci of the same experiment (upper panels on the left). The average pixel intensity (grey value) inside an ROI was taken to represent the focus intensity. The background was measured for each cell individually (cell shapes were determined by DAPI staining). For this, the most frequent (modal) grey value of the respective cell was determined which provided nearly identical results to the average grey value of the region without foci (see histogram on the right). The foci intensities were then normalized to the background intensity of the respective cell to account for variations in staining efficiency between different cells and samples. (B) A549 tumor cells treated with BRCA2 siRNA were irradiated with 1 Gy (0.5 h) or 2 Gy (8 h), immuno-stained as in Figure 1A, and focal intensities of γH2AX were measured using ImageJ software. (C) RAD51 foci were analyzed in G2-irradiated A549 tumor cells. Cells were treated with ATMi 0.5 h prior to or 1 h post IR. Foci numbers from unirradiated cells were subtracted. At least 40 cells were analyzed per data point and experiment (mean ± SEM from ≥3 experiments). P values were obtained by t-test and represent a comparison of all cells analyzed in the indicated cell populations (***: p<0.001). (PDF) [file pgen.1003667.s001.pdf]

A

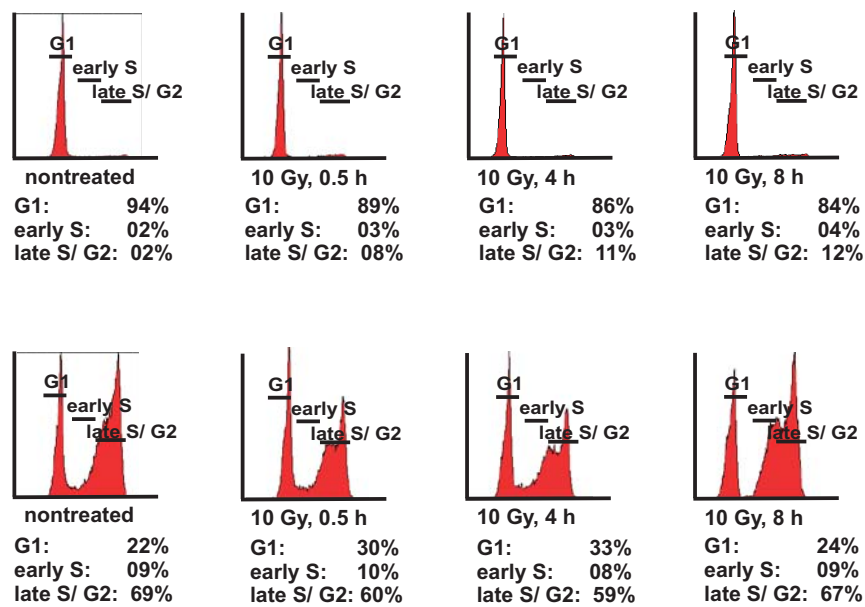

B

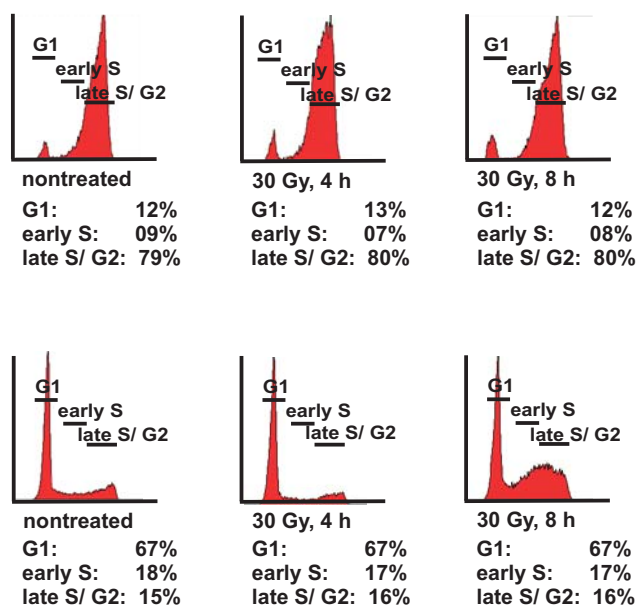

C

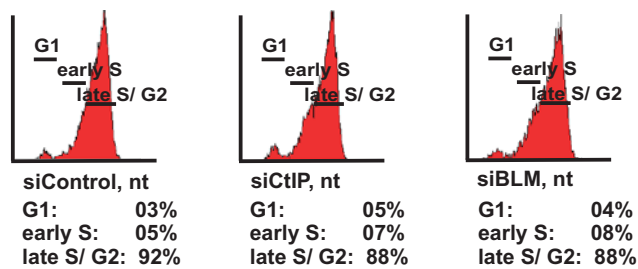

Supplement: Figure S2 — (A) Cell cycle distributions of A549 tumor cells after synchronization in G1-phase by serum starvation (upper panels) or enrichment in G2 phase by double thymidine blocking (lower panels). (B) Cell cycle distributions of HeLa tumor cells after synchronization in G2 phase by double thymidine blocking (upper panels) or without synchronization (lower panels). (C) Cell cycle distributions of HeLa tumor cells after treatment with either CtIP or BLM siRNA and synchronization in G2 phase by double thymidine blocking. (PDF) [file pgen.1003667.s002.pdf]

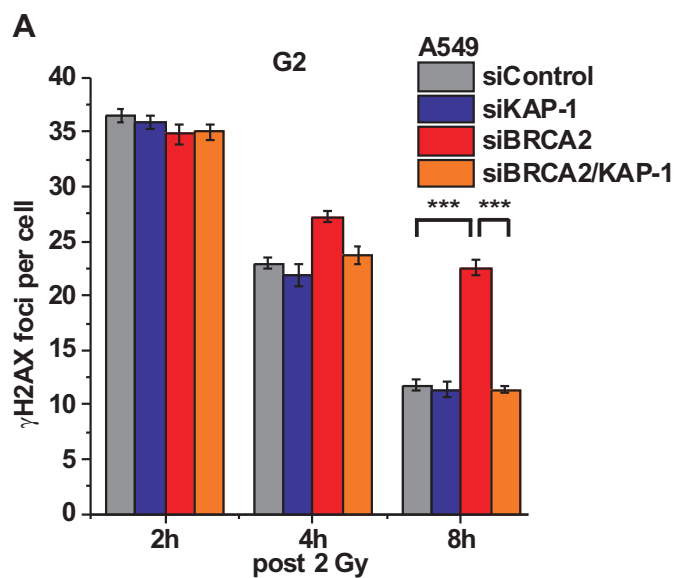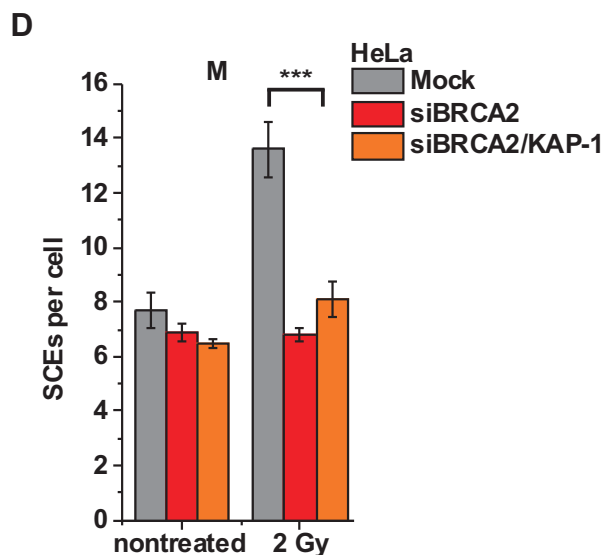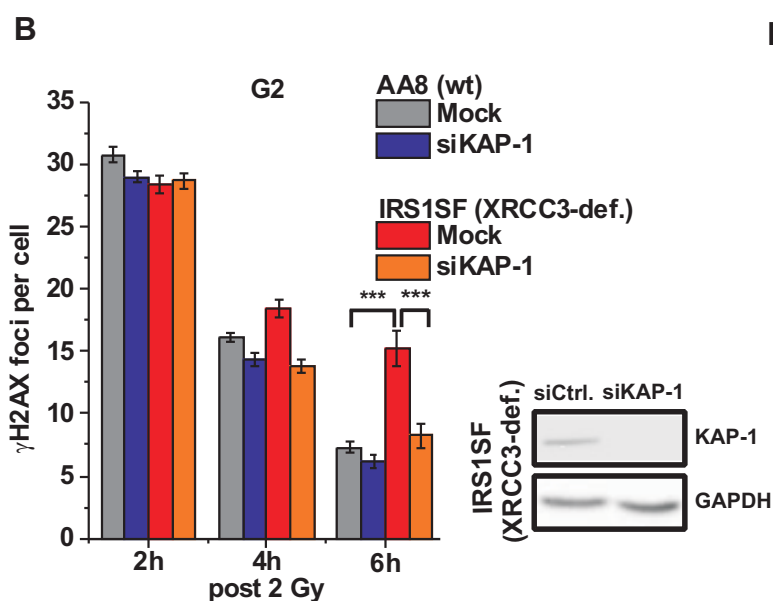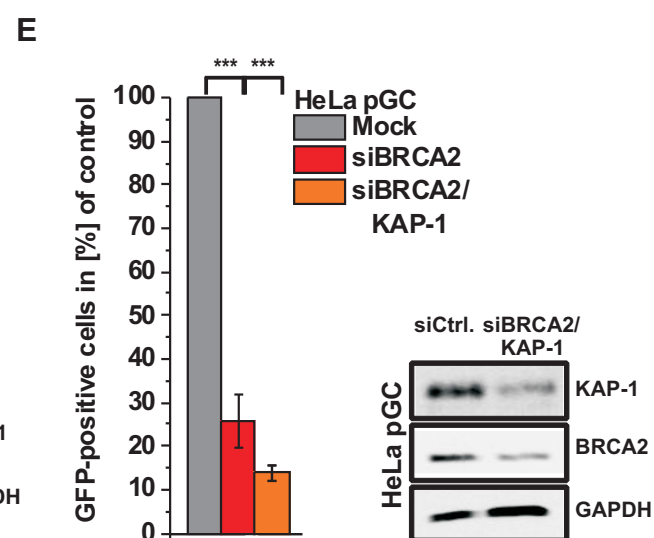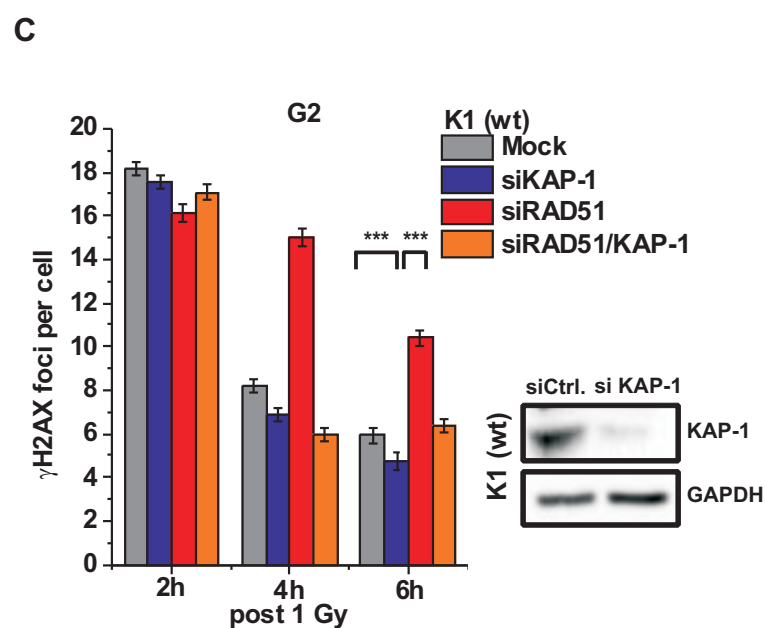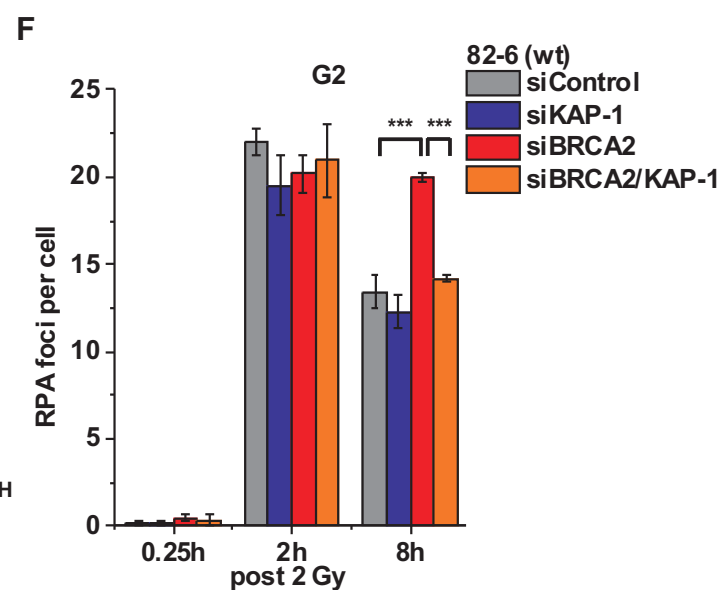

Supplement: Figure S3 — (A) γH2AX foci were analyzed in G2-irradiated A549 tumor cells. (B, C) γH2AX foci were analyzed in G2-irradiated AA8 (wt) and IRS1SF (XRCC3-deficient) (panel B) or K1 (wt) (panel C) CHO cells. In samples treated with RAD51 siRNA, only RAD51-foci-negative cells were analyzed. (D) SCEs in G2-irradiated mitotic HeLa tumor cells at 8 h post 2 Gy. Cells were treated with caffeine and colcemid at 5 h post IR to abolish the G2 checkpoint and collected in mitosis. (E) HR frequencies (gene conversion) after I-SceI expression in HeLa pGC cells carrying an integrated GFP reporter system. (F) RPA foci were analyzed in G2-irradiated 82-6 hTert (wt) human fibroblasts. Foci numbers from unirradiated cells were subtracted. At least 40 cells were analyzed per data point and experiment (mean ± SEM from ≥3 experiments). P values were obtained by t-test and represent a comparison of all cells analyzed in the indicated cell populations (***: p<0.001). (PDF) [file pgen.1003667.s003.pdf]

**A**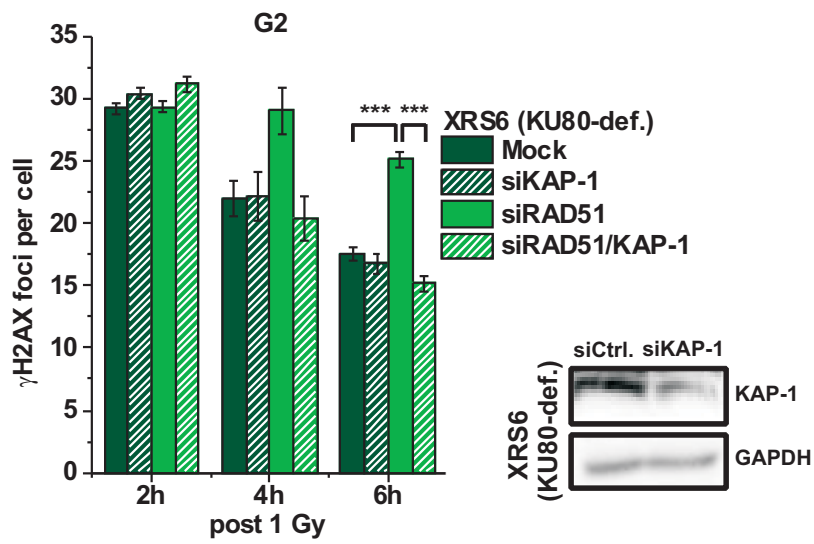**B**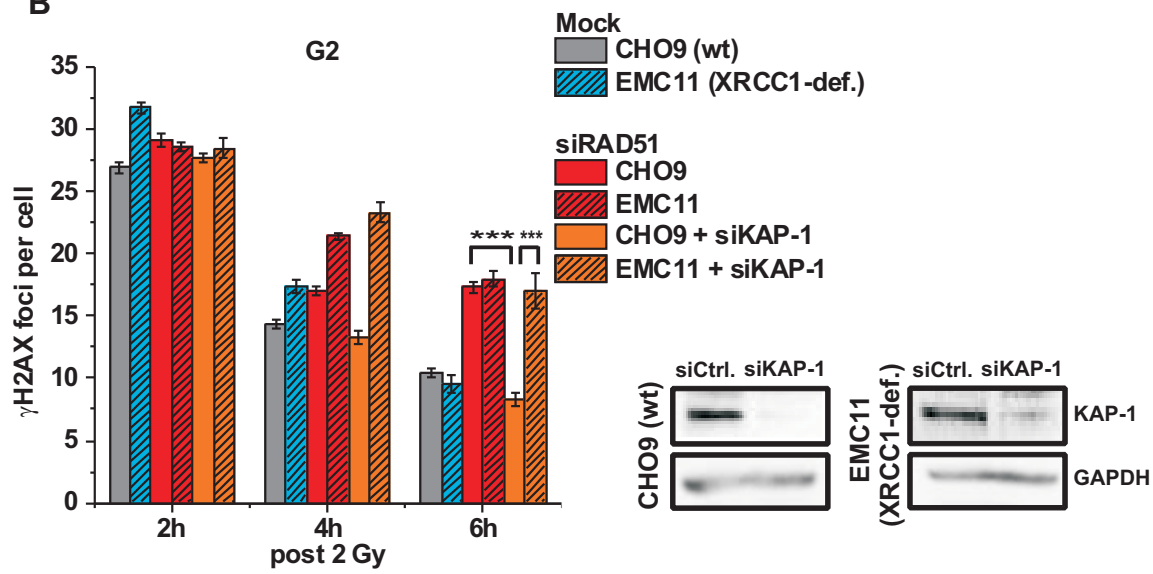

Supplement: Figure S4 — (A) γH2AX foci were analyzed in G2-irradiated XRS6 (KU80-deficient) CHO cells. (B) γH2AX foci were analyzed in G2-irradiated CHO9 (wt) and EMC11 (XRCC1-deficient) CHO cells. In samples treated with RAD51 siRNA, only RAD51-foci-negative cells were analyzed. Foci numbers from unirradiated cells were subtracted. At least 40 cells were analyzed per data point and experiment (mean ± SEM from ≥3 experiments). P values were obtained by t-test and represent a comparison of all cells analyzed in the indicated cell populations (***: p<0.001). (PDF) [file pgen.1003667.s004.pdf]
